# Supplementary material for: Time to publish? Turnaround times, acceptance rates, and impact factors of journals in fisheries science
Source: PLoS One. 2021 Sep 23;16(9):e0257841. doi: 10.1371/journal.pone.0257841 (PMC8459974; doi:10.1371/journal.pone.0257841)
Supplement: Attachment — Submitted filename: my comments.docx [file pone.0257841.s002.docx]

*Summary*

The manuscript presents a concise analysis of turnaround times for journals in fisheries science. The paper is well written and logically structured, and frankly enjoyable to read.

I have no outright major concerns. Most of my comments and questions regard formatting conventions and clarity, particularly in the presentation of the methods and results.

**Title** is clear and appropriate.

**Abstract** is well written.

Line-specific comments:

31: Aarssen et al. Unless “Aarssen, Trengenza (4)” is acceptable formatting for PLOS One, this looks like only two authors are on this manuscript (or a typo).

35: Mulligan et al. Check (and correct) this formatting throughout the manuscript.

38: May be worth mentioning that Nguyen et al. (7) surveyed researchers in conservation biology. In fact, this may be useful context for the other statistics cited here (like you mentioned for Aarssen and Tregenza (4)).

61-62: Why wouldn’t cross-discipline comparisons “be apt”? Maybe more specifically, this is beyond the scope of your objective, which is to provide a sort of fact sheet of turnaround times for authors in fisheries sciences to consider when deciding on a journal?

74: Why only 2010-2020? Electronic versions were available in the 2000s as well.

76: Again, why the >400 threshold? It may be clearer to bullet point or tabulate all the specific criteria used in your query to create your dataset.

90: I thought papers since 2010 were considered, but this says papers published since 2018? What is the actual criterion (and sample size) for analysis?

132: Was this demonstrated or directly observed (that “many journals offered leniency…”)? Perhaps this is better posited as a hypothesis or suspected phenomenon based on your own observations.

137: Times in review were shorter during the beginning of the pandemic than before? This seems counterintuitive to the line of reasoning implied in the previous sentences (journal leniency in deadlines leading to extended turnaround times). Please clarify or elaborate on your hypothesis.

147-149: The topic sentence is somewhat misleading for the paragraph (e.g. IF is flawed, but I use it anyway). Suggest introducing IF as most widely used and easily accessed metric, and moving the current topic sentence to later in the paragraph as a caveat.

171-172: This makes me wonder if there is a universal standard/definition for how each journal calculates acceptance rates. Is it the number of papers accepted out of the total number of submissions? Does it account for resubmissions that were invited after an initial rejection (or more common practice nowadays)? Suggest stating this caveat to these statistics.

189-190: Could you be more precise than “often”? For example, how many journals had the middle 50% span at least 100 days?

200-201 & Fig. 4: “Negative”? First, Figure 4A shows acceptance rate VS proportion peer review. Second, if looks like there would be a positive correlation between these two values if any relationship. I see the color scale reflects Impact Factor, which looks to have a negative correlation with acceptance rate. My dissection digression aside, I suggest directly plotting the two quantities (acceptance and desk rejection rate, or impact factor with rejection and acceptance rates separately) to make these correlations apparent to the reader.

203-204: It is convention to say the dependent variable against the independent variable so “overall acceptance rate plotted against time to first decision”, not the other way around.

234-238: Ahh, I believe I have my answer to 171-172. Perhaps this text is better placed at that location as a caveat (in the Methods).

Fig. 6: Again, advise changing to “Median time to publication versus impact factor”

Fig. 7: I believe I understand what this figure is trying to convey, but it is still unclear as currently formatted. To better discern any trends or long-term changes, I have the following suggestions

- Plot a different colored horizontal line at 1.0 (the baseline)
- Perhaps plot the percentiles (as a ribbon plot?) to clearly summarize how key aspects of the publication time distribution changes over this time frame (e.g. median, inner 50^th^ and 95^th^)
- Or plot the median, smoother, moving average, or linear fit over these lines and the baseline
