# Supplementary material for: Time to publish? Turnaround times, acceptance rates, and impact factors of journals in fisheries science
Source: PLoS One. 2021 Sep 23;16(9):e0257841. doi: 10.1371/journal.pone.0257841 (PMC8459974; doi:10.1371/journal.pone.0257841)
Supplement: Attachment — Submitted filename: Response to Reviewers.docx [file pone.0257841.s003.docx]

Editor:

My remaining comment on the manuscript is that some discussion should be made on the limitations of using the journal impact factor as a proxy for individual articles' quality. Although this is briefly mentioned in the methods (L147-149), I suggest dedicating a paragraph in the discussion to the drawbacks of impact factors. A good location may be to add text between the 2nd and 3rd or 3rd and 4th paragraphs.

I appreciate the editor’s comment and suggestion regarding the discussion on impact factor. I agree that the use of impact factor is flawed, and that the limitations deserved more attention in this paper. To that end, and as suggested, I have included a new paragraph (with several new citations) in the Discussion. The new paragraph may be found beginning on line 275, and reads:

*In this study, I treated impact factor as a proxy for the quality of individual journals. While impact factor is often still used in this way [22], its limitations are well-documented by authors across many disciplines [e.g., 25, 26, 27]. For instance, the calculation of how many “citable” documents a single journal has produced is often dubious, as this may or may not include errata, letters, and book reviews depending on the publisher [28]; misclassification can inflate or deflate a given journal’s impact factor, and the rate of misclassification may depend on the individual journal’s publishing paradigm [29]. Alternatives to impact factor, such as SCImago Journal Rank (SJR) and H-index, have been proposed and may in some cases be more valid metrics of journal prestige or quality [30, 31]. Comparison of these bibliometrics among journals in fisheries was beyond the scope of this paper, and I elected to use only impact factor given its ubiquity and despite its known disadvantages.*

Reviewer #1: In this study, the author compiled a number of publication metrics for a variety of fisheries or fisheries-adjacent journals and a select few other journals. Such metrics are relevant to the decision-making of authors of original research articles in journal selection. The study and compiled dataset therefore serve an immediately practical and useful service to anyone in fisheries and related fields trying to publish. The author also considered the relationships between the various collected metrics ant the COVID-19 pandemic. The compiled synthesis and analysis yielded interesting results, including some such as the limited COVID-19 impacts that defied the author’s presented hypothesis and my own expectations. The data collection process could serve as a guide to replicate the work on a wider scale or in a different field, leaving a template for others who may be interested in conducting such an effort. The study highlights the need for journals to report some of the metrics compiled themselves for the purposes of transparency.

The different decisions made by the author are clearly outlined and explained in the methods, so while I may have made slightly different decisions were I to conduct the work, I support the author’s decisions and subsequent analyses. The reasoning behind the statements in the discussion and introduction seemed largely reasonable and the figures and table all supported the results and objectives of the paper. The limitations of the study are clearly outlined, particularly in some of the speculations included in the discussion, which I appreciated. The inclusion of the raw table and Table 1 is also a wonderful example of data transparency.

I am grateful to the reviewer for their thoughtful comments on this manuscript.

I do have one general concern with the paper, which is that the author’s statements in the introduction and discussion are frequently not supported by citing literature. Many statements, such as almost all of the two paragraphs from lines 45-58, did not have citations to support various statements. It is possible the author made these statements based on their own experience working through their data collection, but if that were the case, I would like to see that more clearly stated or that some of the statements in the introduction should be moved to the discussion. This could also be alleviated by adding more citations to support various statements or at the very least by adding examples of journals that have the different policies or behaviors described by the author.

I thank the reviewer for raising this point. I have added a “personal observation” to one statement (L46) and to another I have cited a “personal communication” from the Editor in Chief of ICES J Mar Sci., one of the most impactful journals in this field (L 55). To the second paragraph in this section, only one personal observation was appropriate in my opinion (L59).

I have also identified more specific changes I would like to see made, outlined below. “L” means line numbers in the manuscript. I do not think that any of these issues deserve the article being rejected, just that the recommended revisions would improve the final published paper were it accepted.

L 35, 48, and elsewhere: The author is inconsistent about how they refer to authors from studies they are citing (e.g., Mulligan, Hall vs. Lewallen and Crane). I could not find PLoS ONE’s format guidelines for this situation, but I would prefer an “and” instead of a “comma.” Regardless, the authors should fix this throughout so it is at least consistent.

I am appreciative that the reviewer noticed this inconsistency and have rectified the citation format throughout the paper.

L 78: I appreciate the use of examples throughout this paragraph and wonder if more examples to some of the sentences in the introduction and discussion such as L 46 “Some journals…” to help contextualize the different assertions made and give readers a place to go to for examples if needed.

I thank the reviewer for this comment, and wanted to be delicate about “calling out” specific journals in this paper especially when the statement(s) could be construed to be critical. That said, I have added an additional example in L 47.

L 81: A comma should follow any use of e.g. or i.e. so this should be corrected throughout.

I thank the reviewer for this comment, and have added commas to all uses of i.e. and e.g. throughout the paper.

L 96-97, 113, 115-121, and elsewhere: The authors should remain consistent about the hyphens used in defining terms (e.g., time-to-acceptance, time-to-publication, time to first decision). The hyphens also disappear from some terms after they are defined as having them and this should also be kept consistent. I personally like the hyphened versions better but would accept either provided it was consistent throughout.

I thank the reviewer for noticing this inconsistency, and have modified the text to include the hyphenated versions throughout the paper. I have also made this change on figure axes.

L 137: An “en” dash should be used for date ranges as is done on L 139, 140

I have made this correction. Thank you.

L 147: If an acronym is going to be used for impact factor, it should be introduced and used after its first appearance in the main text when it’s mentioned in the introduction, not here in the methods. The acronym should then be used throughout, which it is not really outside of this paragraph. Also, impact factor is capitalized here when it is usually lower case in the manuscript, so this should also be kept consistent. I personally think impact factor could be lowercase and not acronymized throughout but would accept another option as long as it was consistent.
I thank the reviewer for noticing this inconsistency and have decided to go with “impact factor” throughout the paper. I have made these changes.

L 151-154: This definition is a little difficult to follow the way it is written out. Maybe it would be clearer if this were broken up into two sentences or if the order of some phrases were rearranged, but I’m not sure how much that would help.

I have attempted to clarify the definition of impact factor. The new text reads: *Impact factor is calculated as the number of citations received in a given year by all articles published in that journal during the previous two years, divided by the number of articles published in that journal during the previous two years.* L160.

L 169: I believe it should be written as “Editors-in-Chief.”

I thank the reviewer for this catch and have made this change.

L 173-174: Change sentence to read “Such cases did not differ in these figures by more than 10%.” or alternatively “No such cases differed in these figures by more than 10%.”

I thank the reviewer for this suggestion and have elected for their first option.

L 203: While I believe I am correct in assuming that these 48 journals were part of the group of 60 journals with acceptance rates collected, it would be nice if that could be stated more clearly in the sentence, such as “Of the 60 journals with overall acceptance rate information, I obtained….”

I thank the reviewer for this comment and have made this change.

L 204-205: Change to “…these variables; however, journals…”
I thank the reviewer for this comment and have made this change.

L 219-221: This should be mentioned in the methods as well as here.
I agree with the reviewer that this is an important point, and have added a sentence in the Methods (L103) that states *“For date published, I used the earliest date after acceptance, i.e., if “date published online” and “date published in an issue” were both provided, I used only “date published online.””*

L 254: Change to “editor-in-chief”
I thank the reviewer for this comment and have made this change.

L284-287: This paragraph is only 2 sentences long and I think could easily be combined with the paragraph above it.

I thank the reviewer for this comment and have made this change.

Figures 1-6: Since colors were used in the figures, I would like to see them consistent so that one color is associated with a particular metric throughout all of the figures as opposed to green, then pink just being the default for all metrics. If the author chose to not use color, then using black then gray throughout would be an alternative option to multiple colors. While I was unable to confirm this because it is dependent on the exact shading, I also suspect the green and pink combination may be challenging colors to differentiate for colorblind readers. The author could avoid this potential issue by using colorblind friendly palettes, as are available and easily accessible, particularly in R if that’s how the figures were made.

I appreciate this comment from the reviewer, but have elected to retain the color scheme. I chose these colors to match the online interface of the journal (PLOS One). If the editor requests, I will use a different color scheme.

Figure 1: Since this figure is made up of two separate graphs, not in a paneled style, I think the graphs should be broken up into Figure 1A and Figure 1B.

I thank the reviewer for this comment and have made this change.

Figure 3: The legend reads “times to publication” but the axis in the figure reads “days from submission to publication” and I think these should be made consistent.
I thank the reviewer for this comment and have made this change to the caption.

Figure 4: While “peer review” as a noun does not need hyphens, “peer-reviewed” is an adjective and therefore does need hyphens so this should be corrected here and throughout if there are other similar instances.
I thank the reviewer for catching this and have made changes in this figure and the caption, as well as one location in the manuscript text.

Figure 7: I think this is the only instance where median is placed in parentheses as opposed to in front of the particular metric, so I think this should be changed to read “Median monthly publication time….”

I thank the reviewer for this comment and have made this change.

Figure 4: Here, the axis reads “Time to First Decision (days),” but the other axes have “days” outside parentheses, so I think that should be kept consistent.

I thank the reviewer for this comment, but have not made a change, since figure 4 is the only figure that uses time-to-first-decision. Unfortunately, I do not have information on whether these values are means, medians, or some other summary statistic as they are provided by the journals. Therefore I cannot change the axis label to (e.g.) “Median time-to-first-decision.” I have retained the original axis label here, but will reconsider on the advice of the editor.

Figure 7: The y axis should be all capitalized to be consistent with the other axes.
I thank the reviewer for this comment and have made this change.

Figures 1-7 and Table 1: I don’t think the author should use the term “fisheries and aquatic sciences” to describe the journals included. Aquatic sciences could include a much wider variety of topics, from aquatic plant physiology to oceanography, that would not have been included in this study. Perhaps “fisheries and related topics” may be more accurate or something similar. This phrase is also only used in the legends, not anywhere else in the paper, as another reason why it should be altered.

I thank the reviewer for this comment and have made this change.

*Summary*

The manuscript presents a concise analysis of turnaround times for journals in fisheries science. The paper is well written and logically structured, and frankly enjoyable to read.

I have no outright major concerns. Most of my comments and questions regard formatting conventions and clarity, particularly in the presentation of the methods and results.

**Title** is clear and appropriate.

**Abstract** is well written.

Line-specific comments:

31: Aarssen et al. Unless “Aarssen, Trengenza (4)” is acceptable formatting for PLOS One, this looks like only two authors are on this manuscript (or a typo).

I thank the reviewer for this comment and have made this change throughout.

35: Mulligan et al. Check (and correct) this formatting throughout the manuscript.

I thank the reviewer for this comment and have made this change throughout.

38: May be worth mentioning that Nguyen et al. (7) surveyed researchers in conservation biology. In fact, this may be useful context for the other statistics cited here (like you mentioned for Aarssen and Tregenza (4)).

I thank the reviewer for this comment and have made this addition.

61-62: Why wouldn’t cross-discipline comparisons “be apt”? Maybe more specifically, this is beyond the scope of your objective, which is to provide a sort of fact sheet of turnaround times for authors in fisheries sciences to consider when deciding on a journal?

I thank the reviewer for this comment and have added a sentence to clarify: *Moreover, my goal in this study is to provide field-specific information, and data on journals in other disciplines was beyond that scope.*

74: Why only 2010-2020? Electronic versions were available in the 2000s as well.

I thank the reviewer for this comment, and note that this time range was used in the journal selection criteria only, and papers from which publication histories were obtained were restricted to ~2018-present since within-journal paradigms are known to change over time. The 2010-2020 selection criteria was used because I wished to make my journal list as relevant as possible to the modern author.

76: Again, why the >400 threshold? It may be clearer to bullet point or tabulate all the specific criteria used in your query to create your dataset.

I have clarified why >400 was used as a selection criteria in this section. New text reads: *This threshold was used because it emerged as a natural break in the list of journals.*

I appreciate the suggestion for a bulleted list, but have elected not to include one at this time as the selection criteria were very few. If the editor wishes that I include a bulleted list, I will acquiesce.

90: I thought papers since 2010 were considered, but this says papers published since 2018? What is the actual criterion (and sample size) for analysis?

I thank the reviewer for this comment. As mentioned in a prior response, 2010-2020 was used when selecting journals for inclusion, but only papers published from 2018-present were included in the analysis. The data on date ranges (and sample sizes) used for analysis are available in Table 1.

132: Was this demonstrated or directly observed (that “many journals offered leniency…”)? Perhaps this is better posited as a hypothesis or suspected phenomenon based on your own observations.

The reviewer brings up a good point. I have changed “many” to “some” and cited this statement as a pers. obs.

137: Times in review were shorter during the beginning of the pandemic than before? This seems counterintuitive to the line of reasoning implied in the previous sentences (journal leniency in deadlines leading to extended turnaround times). Please clarify or elaborate on your hypothesis.

I state that the hypothesis is that review times will be different, not necessarily shorter. The citation in this section (Hobday et al.) showed that times at the beginning of the pandemic were indeed shorter - I agree that this is counterintuitive. Some of the confusion in this section likely stemmed from the word “Indeed” which I have eliminated.

147-149: The topic sentence is somewhat misleading for the paragraph (e.g. IF is flawed, but I use it anyway). Suggest introducing IF as most widely used and easily accessed metric, and moving the current topic sentence to later in the paragraph as a caveat.

I appreciate this comment, but based on a major comment from the editorial staff I feel that I must retain the caveat regarding the unreliability of impact factor as the topic sentence in this paragraph. I will defer to the editor if they wish the topic sentence to be changed.

171-172: This makes me wonder if there is a universal standard/definition for how each journal calculates acceptance rates. Is it the number of papers accepted out of the total number of submissions? Does it account for resubmissions that were invited after an initial rejection (or more common practice nowadays)? Suggest stating this caveat to these statistics.

I thank the reviewer for this thoughtful comment. I have added a caveat, as suggested, on line 183. *It is possible that there are discrepancies in the calculation of acceptance rates, e.g., resubmissions may be tabulated differently among journals. I made no attempt to account for these potential differences in the present study.*

189-190: Could you be more precise than “often”? For example, how many journals had the middle 50% span at least 100 days?

I agree that this is imprecise, however I have elected not to make any changes to this section as per-journal information is available in the cited figure (Figure 3). Providing additional statistics such as this would, in my opinion, bog down an already data-heavy results section.

200-201 & Fig. 4: “Negative”? First, Figure 4A shows acceptance rate VS proportion peer review. Second, if looks like there would be a positive correlation between these two values if any relationship. I see the color scale reflects Impact Factor, which looks to have a negative correlation with acceptance rate. My dissection digression aside, I suggest directly plotting the two quantities (acceptance and desk rejection rate, or impact factor with rejection and acceptance rates separately) to make these correlations apparent to the reader.

I appreciate the reviewer’s comment, and agree that this section was very confusing. I have edited the text for clarity. New text in this section reads: *There was a weak positive correlation between this value and rate of peer review, implying that rates of the two types of rejections are not independent (Figure 4A).*

203-204: It is convention to say the dependent variable against the independent variable so “overall acceptance rate plotted against time to first decision”, not the other way around.

I thank the reviewer for this comment and have made this change.

234-238: Ahh, I believe I have my answer to 171-172. Perhaps this text is better placed at that location as a caveat (in the Methods).

I have elected to mention this issue in both locations; as mentioned above, new text in the Methods begins in L 183.

Fig. 6: Again, advise changing to “Median time to publication versus impact factor”

I thank the reviewer for this comment and have made this change.

Fig. 7: I believe I understand what this figure is trying to convey, but it is still unclear as currently formatted. To better discern any trends or long-term changes, I have the following suggestions

- Plot a different colored horizontal line at 1.0 (the baseline)
- Perhaps plot the percentiles (as a ribbon plot?) to clearly summarize how key aspects of the publication time distribution changes over this time frame (e.g. median, inner 50^th^ and 95^th^)
- Or plot the median, smoother, moving average, or linear fit over these lines and the baseline

I appreciate the reviewer taking the time to comment on this figure, and have added a horizontal line at 1.0. However, I have not otherwise altered this figure, as I believe transforming these values in any way would change the interpretation of the figure. I am attempting to show the true among-journal variation in this value, and offering smoothing or summary visualizations (such as a ribbon plot) would not give the reader as genuine a look at the population of journals. If the editor suggests additional changes, I will acquiesce.
